# Development of a droplet digital PCR assay for detection of group A porcine rotavirus

**DOI:** 10.3389/fvets.2023.1113537

**Published:** 2023-03-06

**Authors:** Yangkun Liu, Xueying Han, Xinru Zhang, Jiaxing Liu, Lunguang Yao

**Affiliations:** ^1^Henan Provincial Engineering and Technology Center of Health Products for Livestock and Poultry, School of Life Science and Agricultural Engineering, Nanyang Normal University, Nanyang, China; ^2^College of Veterinary Medicine, Northwest A&F University, Yangling, Shaanxi, China

**Keywords:** porcine rotavirus, droplet digital PCR, real-time quantitative PCR, VP6 gene, detection

## Abstract

Group A porcine rotavirus (PoRVA) is an important pathogen of acute enteritis in piglets, which has caused severe economic losses in the pig industry worldwide. A convenient, sensitive and specific diagnosis method is an urgent requirement for the surveillance of the PoRVA circulating in clinical samples. In this study, a novel and convenient droplet digital PCR (ddPCR) for the detection of PoRVA was developed using the conserved region of the VP6 gene. The detection limit of ddPCR was 1.81 ± 0.14 copies/rection, ~10 times greater sensitivity than TaqMan real-time quantitative PCR (qPCR). Both ddPCR and qPCR assays exhibited good linearity and repeatability, and the established ddPCR method was highly specific for PoRVA. The results of clinical sample testing showed that the positivity rate of ddPCR (5.6%) was higher than that of qPCR (4.4%). Therefore, the newly developed ddPCR assay could be widely used in clinical diagnosis of PoRVA infections.

## Introduction

Group A rotaviruses (RVA), which belong to the family Reoviridae, are a major pathogen associated with acute diarrhea and dehydration in many species, including humans, pigs, calves, horses, and dogs (1–4). In pigs, porcine RVA (PoRVA) can cause severe mortality and morbidity in piglets, resulting in severe economic losses to the global pig industry (5). Furthermore, PoRVA are suspected to be transmitted zoonotically between pigs and humans, which may poses a potential threat to human and animal health (6–9). Therefore, it is necessary to investigate the epidemiology of PoRVA in pigs.

To date, several diagnostic methods, including virus isolation (10), enzyme-linked immunosorbent assay (ELISA) (11), RT-PCR (12), RT-qPCR (13), RT-recombinase aided amplification (RAA) (14) and loop-mediated isothermal amplification (LAMP) (15), have been developed for the detection of PoRVA infection. However, these methods suffer from low specificity and sensitivity, or do not allow direct quantification of viral nucleic acid, thus rendering them unsuitable for routine diagnosis in the early stages of viral infection. Therefore, the development of a rapid, simple, and reliable diagnostic method is imperative for detecting PoRVA.

Droplet digital PCR (ddPCR) is an innovative third-generation PCR technology for absolute quantification of nucleic acids without the requirement of a standard curve (16–18). The ddPCR uses the same target-specific primers and fluorescent probe as TaqMan-based qPCR. In ddPCR, the reaction mixture is separated into tens of thousands to millions of water-in-oil droplets prior to massively parallel PCR amplification. At end point, each droplet is classified as positive or negative based on the recorded fluorescence signal, and the fraction of positive droplets is counted to calculate the target copy number in the sample using Poisson algorithms. The ddPCR method has been demonstrated to have higher sensitivity and specificity than qPCR, especially when the quantity of nucleic acids is very low (19). However, no ddPCR assay is currently available for PoRVA molecular detection. In this study, a ddPCR assay was developed for detection and quantification of PoRVA in clinical samples of pig. Furthermore, the sensitivity, specificity and repeatability of ddPCR was compared with qPCR.

## Materials and methods

### Viruses and clinical samples

All viruses used in this study were collected in our laboratories, including porcine RVA (PoRVA), transmissible gastroenteritis virus (TGEV), porcine epidemic diarrhea virus (PEDV), classical swine fever virus (CSFV) attenuated vaccine, porcine reproductive and respiratory syndrome virus (PRRSV), porcine circovirus type 2 (PCV2), and pseudorabies virus (PRV). A total of 135 clinical samples (small intestine contents and feces) of newborn piglets with diarrhea symptoms collected from 24 pig farms located in Henan province of China from September 2019 to July 2022, were subjected to detection by the assay developed in this study.

### Primers and probes design

Twelve *VP6* gene sequences from representative PoRVA strains, including KU739970.1, MK228043.1, EU372799.1, AB924088.1, AB779621.1, KJ482501.1, KY053147.1, KJ482487.1, KC610704.1, JQ343834.1, MH267276.1, and KT820771.1, were retrieved from the GenBank database and aligned using MEGA 6.0 software to identify the highly conserved regions within the *VP6* gene (Supplementary Figure 1). According to the analysis results, the primers and TaqMan probe were designed subsequently (Table 1). All primers and probes were synthesized by GenScript Biotech (Nanjing, China).

**Table 1:** Sequences of primers and probe for assays for PoRVA.

**Primer/probe**	**Sequence (5^′^-3^′^)**	**Length (bp)**	***T*_m_ (°C)**
VP6 F	ATGGAGGTTCTGTATTCATTG	21	51.7
VP6 R	TCACTTAATCAACATGCTTCTA	22	50.8
ddPCR-F	AATATGACACCAGCAGTTGC	20	53.2
ddPCR-R	GACGTACTGATGTCACATTT	20	51.3
ddPCR-P	FAM-CCGCAAGCACAGATTCACAAACTGCA-BHQ1	26	62.1

### Nucleic acid extraction and reverse transcription

All viruses and clinical samples were re-suspended (20%, W/V) in phosphate-buffered saline (PBS, pH 7.4), vortexed and centrifuged at 12,000 × g at 4°C for 10 min to obtain the supernatant. Regardless of whether the genome of the target virus is DNA (PCV2 and PRV) or RNA (PoRVA, TGEV, PEDV, CSFV and PRRSV), viral nucleic acids were extracted from the supernatants using AxyPrep™ Viral DNA/RNA Miniprep Kit (Axygen, Shanghai, China), according to the manufacturer's instructions. Each viral RNA was employed for the synthesis of the first strand cDNA with the AMV reverse transcriptase. The cDNA/DNA was used immediately for amplification or stored at −80°C until use.

### Construction of standard plasmids

A 1,194-bp *VP6* gene fragment of group A rotavirus was amplified by using primers VP6 F and VP6 R (Table 1) and cloned into the pMD18T vector (TakaRa, Dalian, China). The pMD18T-VP6 plasmid was purified by using the Plasmid Mini Kit (OMEGA Biotech, Shanghai, China) according to the manufacturer's instructions and quantified by NanoDrop 2000 spectrophotometer (Thermo Fisher, USA). The number of plasmid DNA copies was calculated using the following formula: amount (copies/μl) = [DNA concentration (ng/μl) × 10^−9^]/(plasmid length in base pairs × 660) × (6.02 × 10^23^). The plasmid was diluted with ddH_2_O to obtain a stock solution containing 10^8^ copies of the standard plasmid per microlitre. The standard curve was generated using 10-fold dilutions (2.0 × 10^0^ – 2.0 × 10^5^ copies/μl) of the standard plasmid.

### Droplet digital PCR assay

The ddPCR was performed with a TD-1 Droplet Digital PCR system (TargetingOne, Beijing, China) following the manufacturer's instructions. In detail, the ddPCR mixture contained 10 μl of 2 × ddPCR Supermix, 800 nM of each primer ddPCR-F/R, 250 nM of ddPCR-P probe, and 2 μl of template, and deionized water was added to a final volume of 20 μl. Then, 20 μl ddPCR mixture and 160 μl oil were loaded onto the droplet generation chip to produce droplets on a drop maker. The droplets were thermally cycled using a protocol of 95°C for 10 min, followed by 40 cycles of 94°C for 30 s and 54–60°C for 1 min, and the temperature ramp rate was set to 1.5°C/s on a T100 thermal cycler (Bio-Rad Laboratories, Inc., USA). Finally, the droplets were detected on a chip reader (TargetingOne, Beijing, China). Positive droplets containing amplified products were distinguished from negative droplets by applying a fluorescence amplitude threshold at the highest point of the negative droplet cluster. The reactions with more than 30,000 accepted droplets per well were used for analysis. The absolute initial copy number of target nucleic acid molecules within each sample was accurately calculated based on the ratio of positive to total droplets using Poisson statistics. To optimize the separation between positive and negative droplets, the optimal annealing temperature for ddPCR was firstly identified by analyzing temperatures of 60, 59, 58, 57, 56, 55, and 54°C, then the primer-to-probe concentration (300:200, 800:250, 500:300, and 400:400 nm) was optimized. The ddPCR assay was performed in triplicate.

### QPCR assay

The same primers and probe were used for ddPCR and qPCR. We conducted the qPCR detection of PoRVA by Bio-Rad C1000 Touch™ Thermal Cycler. The reaction system (20 μl) included: 2 × TaqMan™ Fast Advanced Master Mix 10 μl, 1.6 μl of each reverse and forward primer (10 μM), 0.5 μl of probe (10 μM), 4.3 μl of ddH_2_O, and 2 μl of template. The amplifying process was as follows: 50°C for 2 min, 95°C for 2 min, 40 cycles of 95°C for 20 s and 57°C for 20 s. After the reaction, a standard curve was plotted, and then the specificity, sensitivity and repeatability tests were performed.

### Sensitivity test of ddPCR and qPCR

The pMD18T-VP6 plasmid was 10-fold serially diluted to achieve DNA concentrations from 2.0 × 10^5^ to 2.0 × 10^0^ copies/μl. Two microliter of each dilution was amplified by ddPCR to determine the linearity of the assay. For comparison, the qPCR assay was performed in parallel using the same templates. Next, the detection limit (LoD) of qPCR and ddPCR were determined according to the Clinical and Laboratory Standards Institute (CLSI) guidelines (20). The pMD18T-VP6 plasmid was diluted to concentrations from 50 to 0.1 copies/reaction. Each concentration was tested in 24 replicates, with an additional 20 replicates of TE buffer as the blank control. Probit regression analysis of 95% hit rates was performed with SPSS 25.0 software (SPSS Inc., Chicago, USA).

### Analytical specificity and reproducibility

To evaluate the specificity of the ddPCR assay, genomes of PoRVA and other six common swine viruses (TGEV, PEDV, CSFV, PRRSV, PCV2, and PRV) were used as templates and tested with PoRVA-specific primers ddPCR-F/R and probe ddPCR-P. The nuclease-free water was used as negative control. Specificity testing was performed under the optimized conditions. In addition, we assayed the plasmid pMD18T-VP6 in 10-fold serial dilutions ranging from 2.0 × 10^4^ to 2.0 × 10^2^ copies/μl, and inter-assay and intra-assay repeatability tests were performed in triplicate for each respective sample to assess variability in ddPCR.

### Clinical sample detection by qPCR and ddPCR assays

To assess clinical effects, 135 clinical samples were detected by ddPCR and qPCR. The amplification conditions were as previously described. The positive detection rate of the two methods was calculated to evaluate the sensitivity of the two methods. Each reaction included a negative control and a positive control. For the assessment of the quantitative consistency, quantitative values of each sample were ascertained using the two assays.

### Statistical analysis

Data were presented as the mean ± SD. All statistical analyses and data plotting were performed using GraphPad Prism software (version 5.0; La Jolla, CA, USA).

## Results

### Development of a PoRVA ddPCR assay

**Figure 1:**
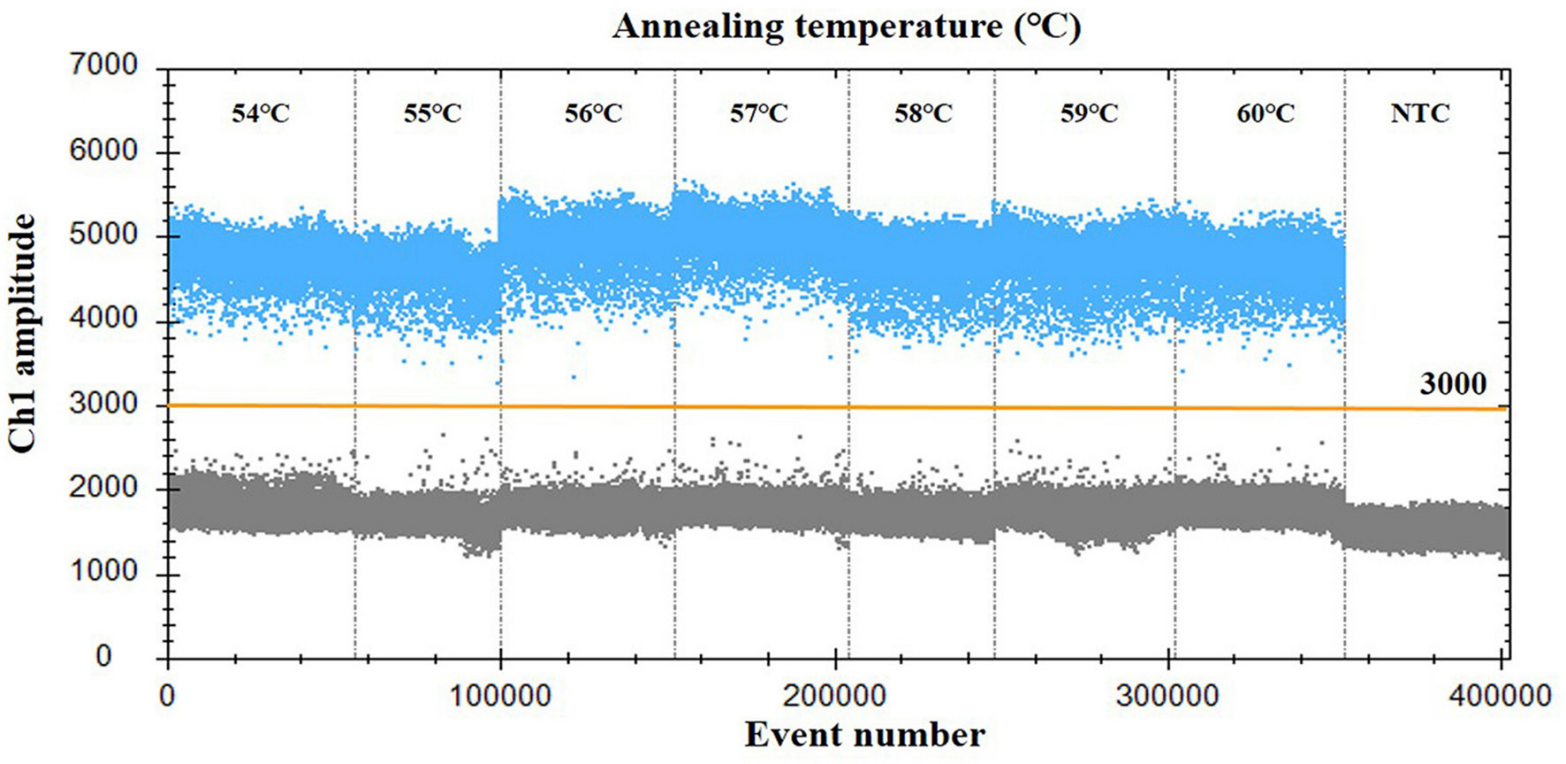
Influence of annealing temperature on the PoRVA ddPCR system. The assay was conducted across an annealing temperature gradient: 54, 55, 56, 57, 58, 59, and 60°C. NTC, no template control.

**Figure 2:**
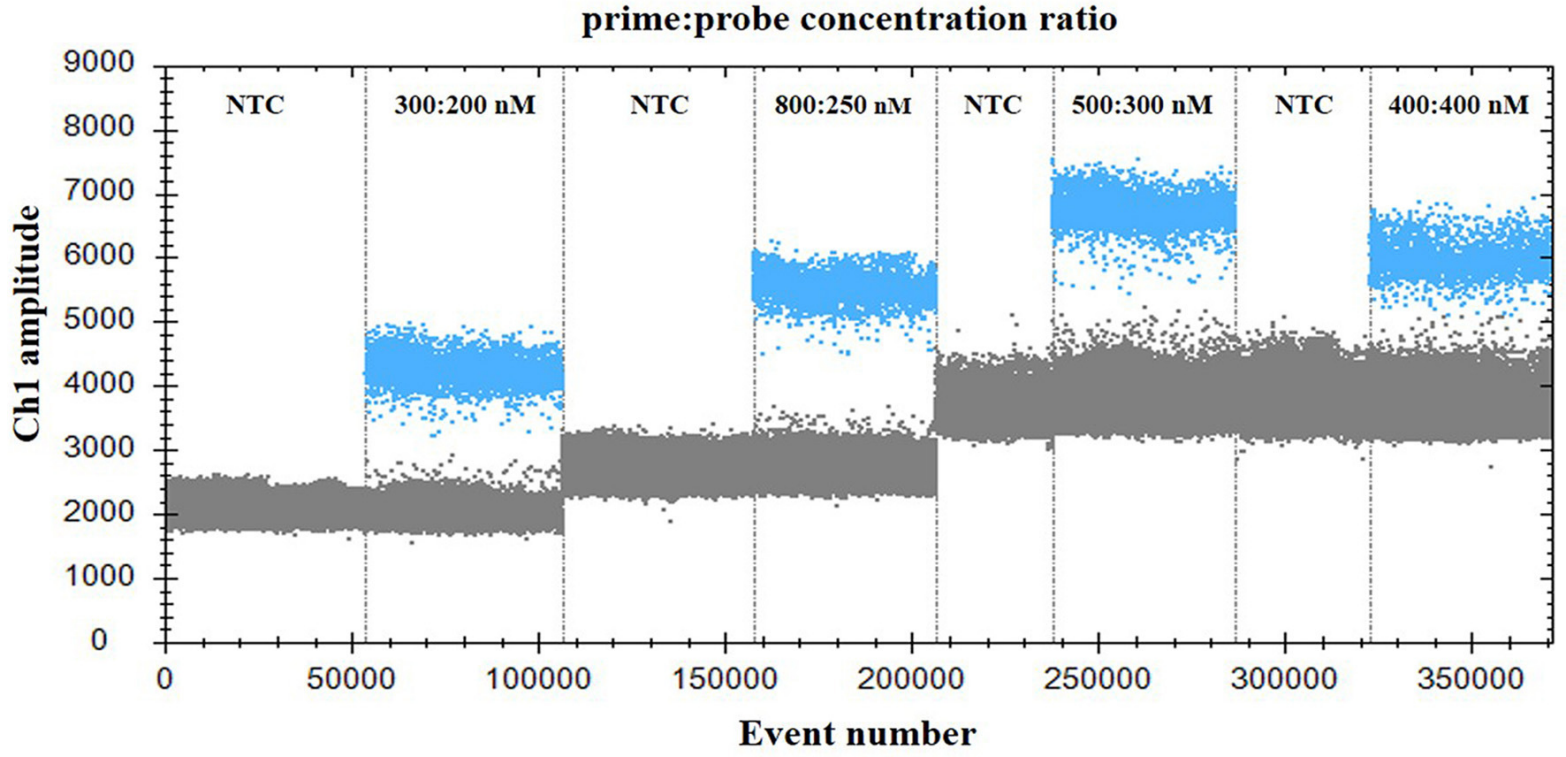
Influence of primer-to-probe concentration ratio on the PoRVA ddPCR system. The assay was conducted across a primer and probe concentration ratio gradient: 300:200, 800:250, 500:300, and 400:400. NTC, no template control.

For optimization of the annealing temperature, the temperature gradients from 54 to 60°C were tested in the ddPCR assay. The results indicated that 57°C provided the greatest difference in the fluorescence signal among the positive and negative droplets (Figure 1), thus it was chosen as the optimal annealing temperature. Next, the primer-to-probe concentration was optimized. The results suggested that, the optimal concentration ratio was 800:250 nM because this ratio of reagents resulted in optimal separation between positive and negative droplets (Figure 2). Therefore, the optimal annealing temperature (57°C) and primer-to-probe concentration (800:250 nm) were selected as the optimized conditions for the subsequent PoRVA ddPCR assay.

### Analytical sensitivity and reproducibility

**Figure 3:**
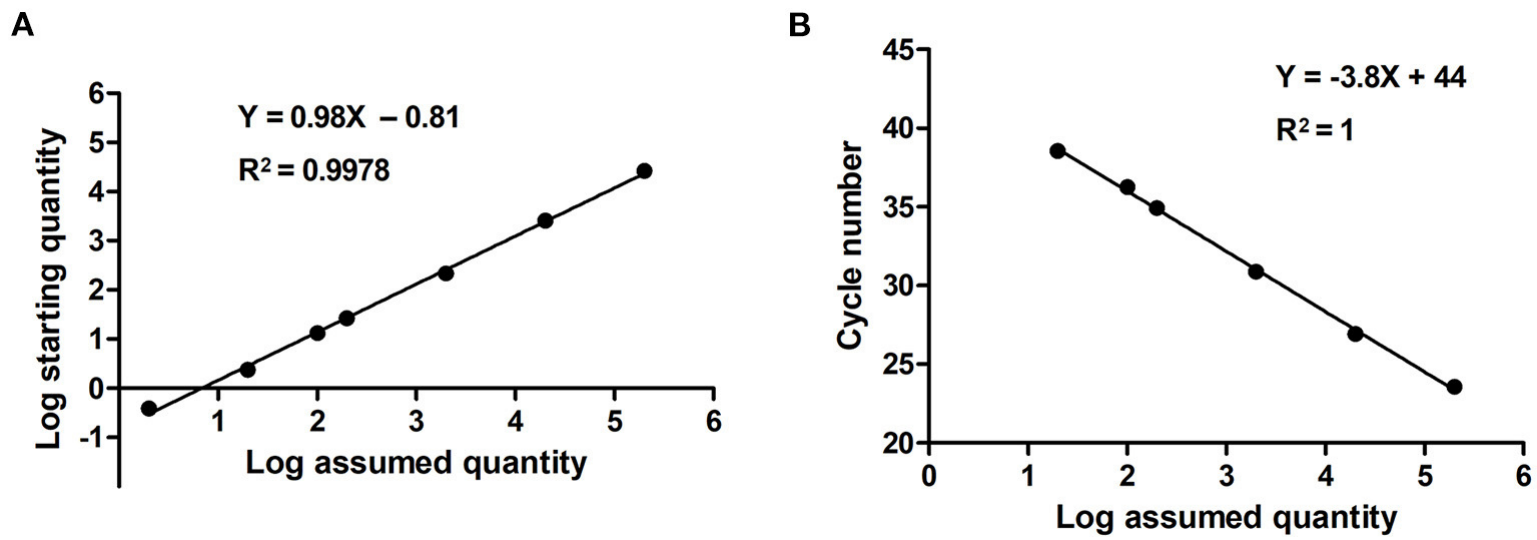
Quantification of serially diluted PoRVA plasmids by ddPCR and qPCR. **(A)** Standard curves PoRVA plasmids constructed by ddPCR. The quantification correlation was obtained by plotting the log assumed concentration against the log starting concentration. **(B)** Standard curves of PoRVA plasmids constructed by qPCR. The quantification correlation was obtained by plotting the quantification cycle value against the log starting concentration.

Serially diluted pMD18T-VP6 plasmids exhibited good linearity in both qPCR and ddPCR assays. In ddPCR, the standard curve exhibited a good linear correlation (*Y* = 0.98*X* – 0.81) with *R*^2^ value of 0.9978 (Figure 3A). In contrast, the standard curve of the qPCR assay was *Y* = −3.8*X* + 44 with *R*^2^ value of 1 (Figure 3B). As shown in Table 2, the detection limit of ddPCR was determined to be 1.81 ± 0.14 copies/rection. By contrast, the detection limit of the qPCR was 18.22 ± 1.23 copies/rection, which was ~10 times higher than that of the ddPCR assay when using a cut-off detection limit of 40 cycles. In the repeatability tests, the intra-assay coefficient of variation (CV) ranged from 2.20 to 4.59%, and the CV of the inter-assay ranged from 1.79 to 5.58% (Table 3). These results showed that the developed PoRVA ddPCR has a good reproducibility.

**Table 2:** Detection limits of quantitative real-time PCR (qPCR) and droplet digital PCR (ddPCR).

**Input of PoRVA plasmid copy number**	**qPCR**		**ddPCR**	
	**Hit rate (positive/total)**	**LoD**	**Hit rate (positive/total)**	**LoD**
50	1.00 (24/24)	18.22 ± 1.23	ND	1.81 ± 0.14
20	1.00 (24/24)		ND	
10	0.83 (20/24)		1.00 (24/24)	
5	0.63 (15/24)		1.00 (24/24)	
2	0.13 (3/24)		0.96 (23/24)	
1	0.00 (0/24)		0.88 (21/24)	
0.5	0.00 (0/24)		0.46 (11/24)	
0.1	ND		0.00 (0/24)	
NTC	0.00 (0/20)		0.00 (0/20)	

NTC, no template control; ND, not detected; LoD, detection limit.

**Table 3:** Robustness and reproducibility analysis of droplet digital PCR (ddPCR).

**Concentration of PoRVA plasmid (copies/μl)**	**Intra-assay variation (robustness)**			**Inter-assay variation (reproducibility)**		
	**Mean (copies/**μ**l)**	**SD**	**CV (%)**	**Mean (copies/**μ**l)**	**SD**	**CV (%)**
2.0 × 10^4^	2,561.9	56.3	2.20	2,552.8	45.7	1.79
2.0 × 10^3^	216.3	8.10	3.75	212.2	8.72	4.11
2.0 × 10^2^	26.5	1.21	4.59	25.3	1.41	5.58

CV, coefficient of variation; SD, standard deviation.

### Analytical specificity of the ddPCR assay

**Figure 4:**
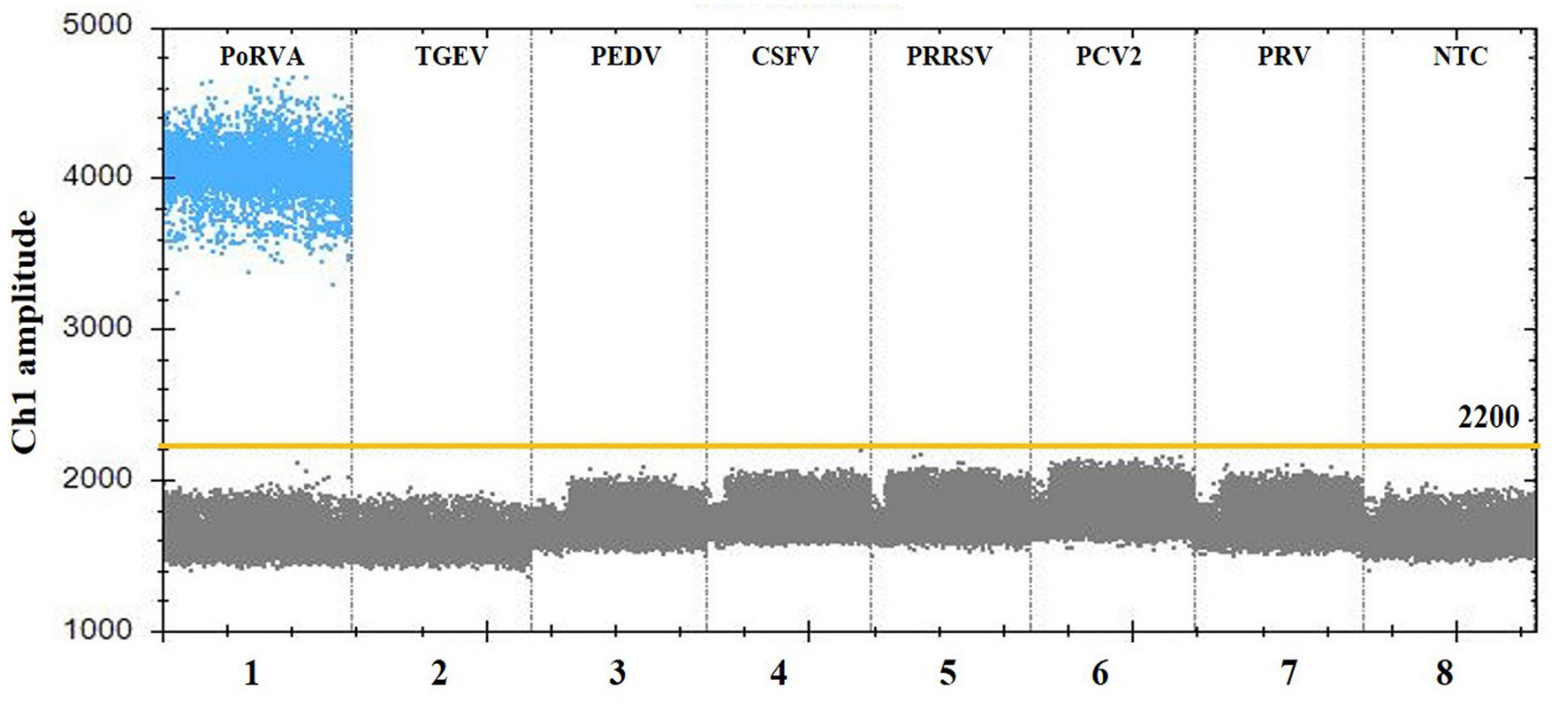
Specificity analysis of the PoRVA ddPCR assay. Lanes 1–8 (divided by vertical black dotted lines): the fluorescence amplitude of PoRVA, TGEV, PEDV, CSFV, PRRSV, PCV2, PRV, and ddH_2_O, respectively.

For the specificity analysis, nucleic acid templates from different pathogens were prepared, including PoRVA, TGEV, PEDV, CSFV, PRRSV, PCV2, and PRV. As shown in Figure 4, only the PoRVA test was positive, while other pathogen tests were negative. The results indicated that this method exhibits high specificity for the detection of PoRVA.

### Clinical sample testing

To further determine the practicality of ddPCR, 135 clinical samples collected from 24 pig farms in Henan Province were evaluated using ddPCR and qPCR. As shown in Table 4, PoRVA was detected with a positive rate of 14.1% (19 of 135) by ddPCR and 11.1% (15 of 135) by qPCR. Four samples detected as negative by qPCR were positive by ddPCR. To exclude false-positive events, the four samples with inconsistent results were retested with the ddPCR assay three times and no template control (NTC) was included in all runs. All of the four samples still tested positive by ddPCR in the presence of valid NTC. To confirm the result of ddPCR, the four fecal samples were first filtered through a 0.22 μm disk filter, and then ultracentrifuged at 41,000 rpm in a sw41 rotor (Beckman) for 90 min. The concentrated samples were treated with DNase I, then amplified using RT-PCR followed by Sanger sequencing, and the sequencing results verified that the four samples were positive for PoRVA. According to these data, ddPCR was found to be more sensitive than qPCR for PoRVA detection in clinical samples.

**Table 4:** Comparison of ddPCR and qPCR sensitivity for PoRVA clinical samples.

		**ddPCR**		**Total**
		**Positive**	**Negative**	
qPCR				
	Positive	15	0	15
	Negative	4	116	120
Total		19	116	135

## Discussion

PoRVA infection is considered an important enteric pathogen in pigs, resulting in significant economic losses due to increased mortality, treatment costs, and reduced weight gain (5, 10, 21). It is worse that PoRVA can be transmitted between humans and pigs worldwide (22–25), posing a high risk to public health. Therefore, continuous surveillance of the PoRVA virus and its epidemic strain is necessary to take appropriate decisions regarding its control. Several etiological and serological methods have been developed for the detection of PoRVA (10–15). Although each of these methods has played an important role in the diagnosis of PoRVA, more sensitive and reliable detection method would provide better diagnostic resources for this virus.

As a third-generation PCR technique, ddPCR has been widely used to detect and quantitative analysis a diverse range of virus (26, 27). Because of enhanced detection sensitivity and highly tolerant to many PCR inhibitors, ddPCR more suitable for the detection of low-level virus genomic copies in host tissue and feces samples, which may contain a high abundance of standard sequences, PCR inhibitors and large numbers of diverse bacteria (28–31). In this study, we established a novel ddPCR method for detection and quantification of PoRVA. This method exhibited high sensitivity and good specificity with low intra- and inter-assay CVs (<6.0%), which indicates that it can provide accurate and reproducible detection results for PoRVA diagnosis. To the best of our knowledge, this is the first report to develop a ddPCR method for PoRVA detection. After optimization of the annealing temperature and primer-to-probe concentration, the ddPCR assay showed a detection limit of 1.81 ± 0.14 copies/rection, which was ~10-fold higher than that of the qPCR assay. In addition, the detection limit of qPCR, RT-RAA and LAMP was five copies/reaction, seven copies/reaction and 100 copies/μl (13–15), which had a lower sensitivity than the ddPCR assay. Higher sensitivity might be conducive to improve the positive detection rate in clinical samples, especially when the samples contain low-level viral nucleic acids. Evaluation of the performance of the ddPCR assay using 135 clinical samples found that four samples tested positive by ddPCR but negative by qPCR, indicating that the ddPCR assay does has a relatively higher PoRVA detection rate than that of the qPCR assay. This result revealed that the ddPCR assay more suitable for the early detection of PoRVA infection and thus help to prevent and control the spread of the virus.

Another advantage is that the ddPCR assay can achieve absolute quantification without the requirement to establish a standard curve. By contrast, qPCR assay can only achieve quantitative detection with the calibration curve produced from serially diluted template, and the calculation of the copy number in samples was dependent on the qPCR Ct values from the standard curve, Therefore, the ddPCR assay was more convenient than qPCR due to calibration curve was unnecessary. Moreover, the ddPCR assay exhibited good specificity and could not detect viral nucleic acids from other important swine pathogens such as TGEV, PEDV, CSFV, PRRSV, PCV2, and PRV, which indicated that the ddPCR method provide a convenient and specific diagnostic and quantification method PoRVA infection.

In addition to the advantages described above, we found that the ddPCR assay could not quantified when the concentration of the DNA sample higher than 2 × 10^5^ copies/μl (data not shown), thereby presenting a small detection range. This finding was consistent with previous reports, in which investigators demonstrated that the upper limit of the sample concentration for ddPCR was 10^5^ copies/μl (32, 33). To overcome this challenge, the high concentration samples should be diluted before test. Nevertheless, ddPCR is a convenient technology for the detection and quantification of the virus, due to the high sensitivity and without the requirement to establish a calibration curve. Moreover, the high concentration samples were still tested positive, which would not affect the result of the qualitatively diagnosis.

In conclusion, a specific, sensitive, and reliable droplet digital PCR assay for the detection of PoRVA was developed and evaluated on the clinical samples. The established ddPCR method exhibits higher sensitivity compared with qPCR, and it was analytically specificity and repeatability, making it a reliable tool for the clinical diagnosis and epidemiological investigation of PoRVA.

## Data availability statement

The original contributions presented in the study are included in the article/Supplementary material, further inquiries can be directed to the corresponding author.

## Ethics statement

The animal study was reviewed and approved by Animal Welfare and Ethics Committee of Nanyang Normal University. Written informed consent was obtained from the owners for the participation of their animals in this study.

## Author contributions

YL and XH designed the study, collected samples, and analyzed the data. YL, XH, XZ, and JL performed the experiments. YL, XH, and LY wrote and revised the manuscript. All authors contributed to the article and approved the submitted version.

## Supplementary material

Supplementary Figure 1 Alignment of primers and probe designed from partial of gene sequences of PoRVA.
