# Supplementary figures and images for: Development of a droplet digital PCR assay for detection of group A porcine rotavirus
Source: Front Vet Sci. 2023 Mar 6;10:1113537. doi: 10.3389/fvets.2023.1113537 (PMC10025470; doi:10.3389/fvets.2023.1113537)

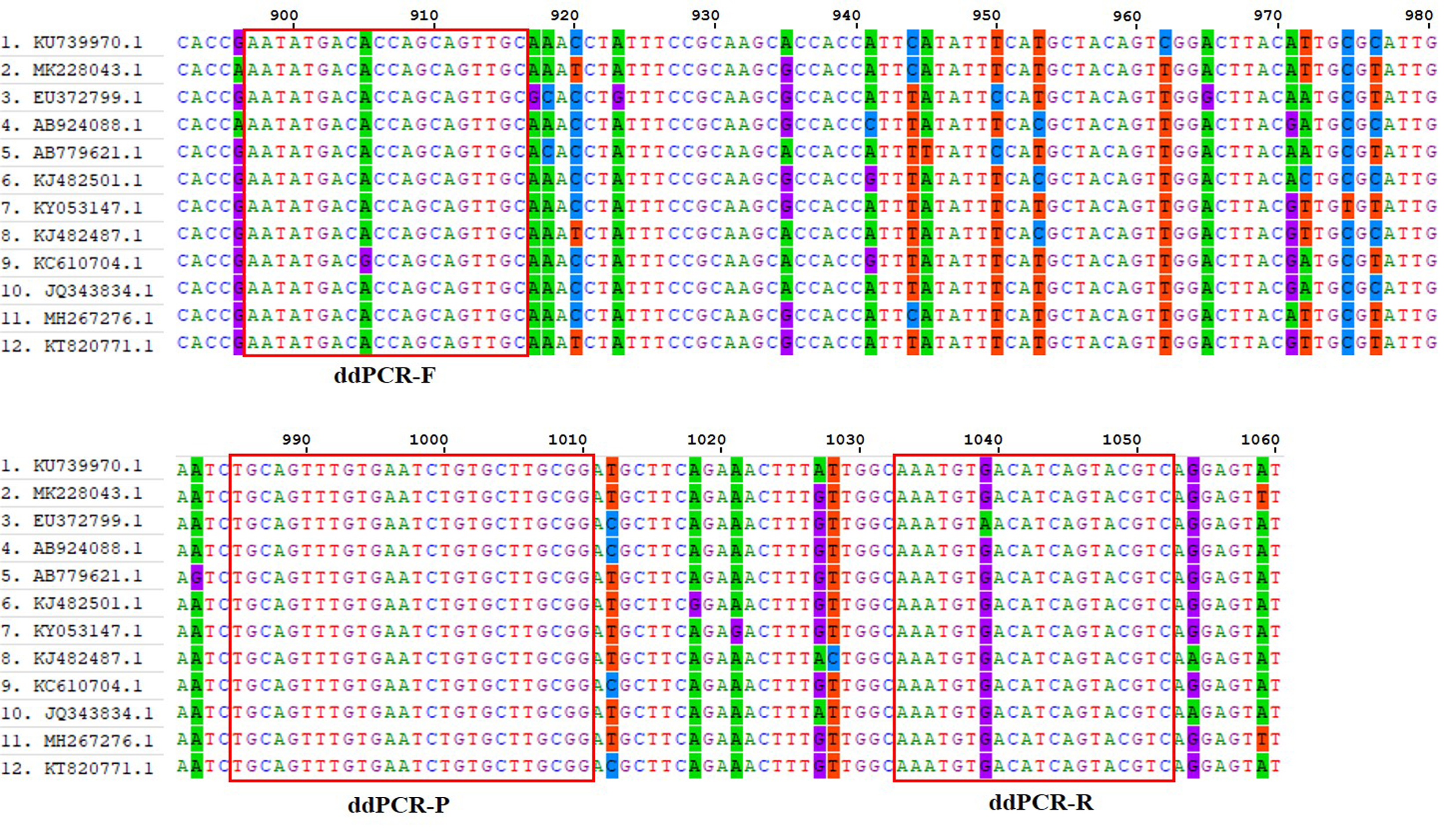

Supplement: Supplementary Figure 1 — Alignment of primers and probe designed from partial of gene sequences of PoRVA. [file Image_1.JPEG]
